# Supplementary material for: Harmine stimulates proliferation of human neural progenitors
Source: PeerJ. 2016 Dec 6;4:e2727. doi: 10.7717/peerj.2727 (PMC5144684; doi:10.7717/peerj.2727)
Supplement: Supplemental Information 1 [file peerj-04-2727-s001.pdf]

Title: Harmine stimulates proliferation of human neural cells

Authors: Vanja Dakic[1,2], Renata de Moraes Maciel[1], Hannah Drummond[1,2], Juliana M. Nascimento[1,3], Pablo Trindade[1], *Stevens K. Rehen[1,2]

[1]D'Or Institute for Research and Education (IDOR), Rio de Janeiro, RJ, Brazil

[2]Institute of Biomedical Sciences, Federal University of Rio de Janeiro, Rio de Janeiro, RJ, Brazil

[3]Department of Biochemistry and Tissue Biology, Institute of Biology, State University of Campinas, Campinas, SP, Brazil

Supplementary information

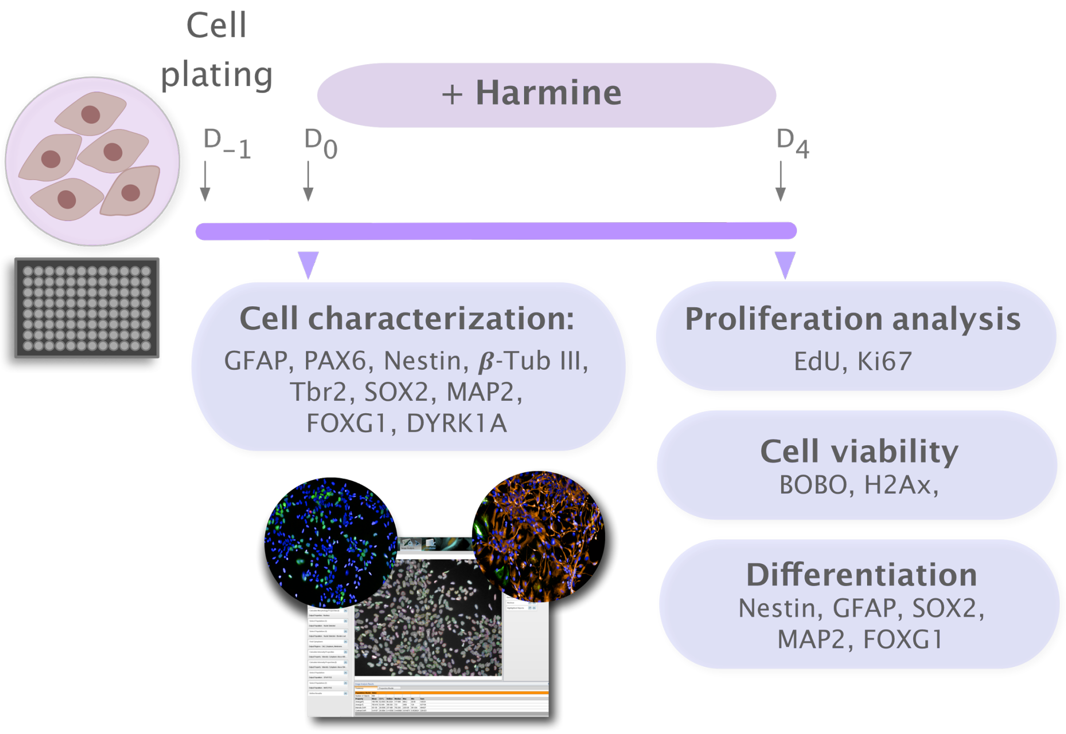

Supplementary figure 1: Scheme for characterization and proliferation experiments.

a

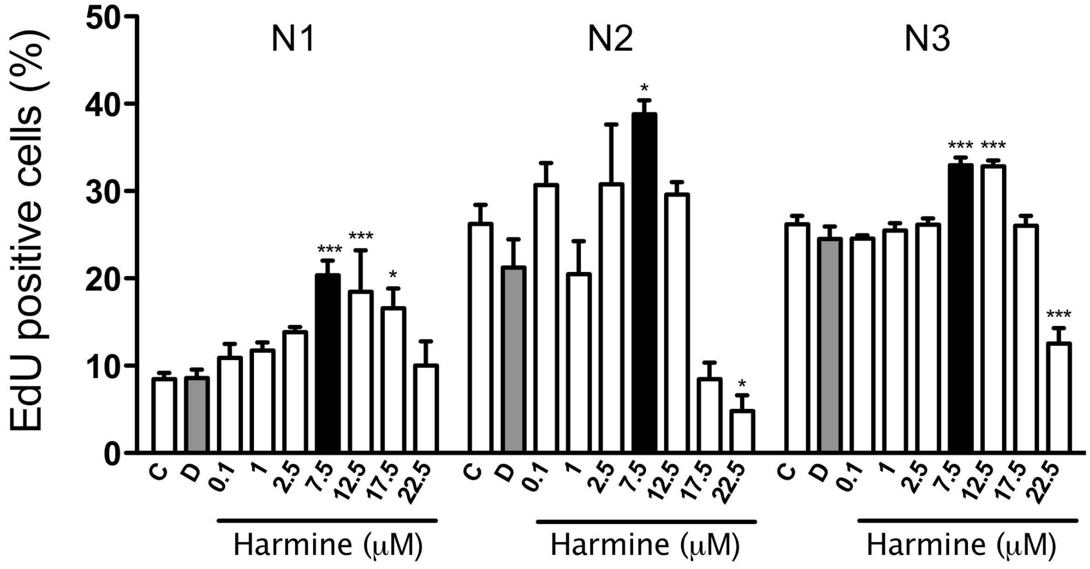

b

	Control DMSO	Harmine (7.5μM)	p
**N1**	8.60±0.94	20.35±1.69	0.0001 to 0.001
**N2**	21.24±3.25	38.79±1.61	0.01 to 0.05
**N3**	24.54±1.4	32.97±0.88	0.0001 to 0.001

Supplementary figure 2: Effect of harmine on cell proliferation. (**a**) Dose-response curve of harmine in 3 different lines of hNPC. (**b**) Table showing mean ± sem and p-value. A minimum of 10,000 hNPCs was counted per condition/per experiment. Data were analyzed by one-way ANOVA with Tukey's multiple comparison test. C - control and D - Control DMSO.

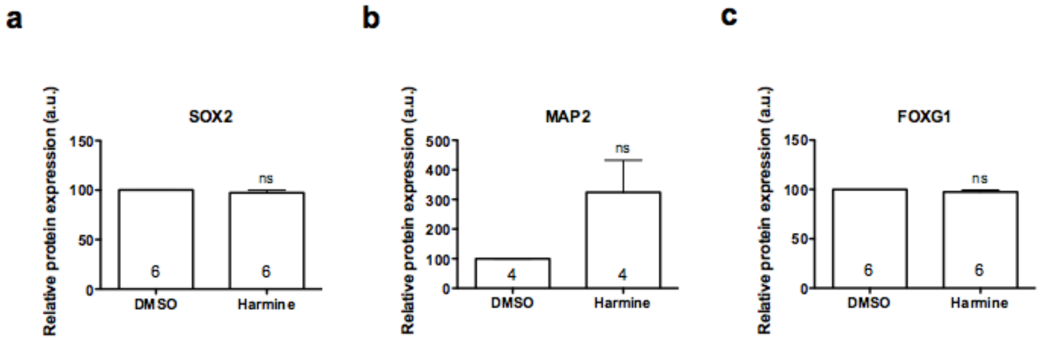

Supplementary figure 3: Quantification of differentiation markers with or without harmine exposure. (**a**) Expression of SOX2 protein relative to DMSO control. (**b**) Expression of MAP2 protein relative to DMSO control. (**c**) Expression of MAP2 protein relative to DMSO control. A minimum of 10,000 hNPCs was counted per condition/per experiment. Data were analyzed by one-way ANOVA with Tukey's multiple comparison test, ns = p>0.1. Values represent mean ± sem. The number inside the bar represents the number of experiments in each group.
